# Rabbit Neonates and Human Adults Perceive a Blending 6-Component Odor Mixture in a Comparable Manner

**DOI:** 10.1371/journal.pone.0053534

**Published:** 2013-01-16

**Authors:** Charlotte Sinding, Thierry Thomas-Danguin, Adeline Chambault, Noelle Béno, Thibaut Dosne, Claire Chabanet, Benoist Schaal, Gérard Coureaud

**Affiliations:** 1 Centre des Sciences du Goût et de l'Alimentation, Dijon, France; INRA-UPMC, France

## Abstract

Young and adult mammals are constantly exposed to chemically complex stimuli. The olfactory system allows for a dual processing of relevant information from the environment either as single odorants in mixtures (elemental perception) or as mixtures of odorants as a whole (configural perception). However, it seems that human adults have certain limits in elemental perception of odor mixtures, as suggested by their inability to identify each odorant in mixtures of more than 4 components. Here, we explored some of these limits by evaluating the perception of three 6-odorant mixtures in human adults and newborn rabbits. Using free-sorting tasks in humans, we investigated the configural or elemental perception of these mixtures, or of 5-component sub-mixtures, or of the 6-odorant mixtures with modified odorants' proportion. In rabbit pups, the perception of the same mixtures was evaluated by measuring the orocephalic sucking response to the mixtures or their components after conditioning to one of these stimuli. The results revealed that one mixture, previously shown to carry the specific odor of red cordial in humans, was indeed configurally processed in humans and in rabbits while the two other 6-component mixtures were not. Moreover, in both species, such configural perception was specific not only to the 6 odorants included in the mixture but also to their respective proportion. Interestingly, rabbit neonates also responded to each odorant after conditioning to the red cordial mixture, which demonstrates their ability to perceive elements in addition to configuration in this complex mixture. Taken together, the results provide new insights related to the processing of relatively complex odor mixtures in mammals and the inter-species conservation of certain perceptual mechanisms; the results also revealed some differences in the expression of these capacities between species putatively linked to developmental and ecological constraints.

## Introduction

From before birth to death, organisms live in a complex chemical world. Strategies exist that allow for the extraction of meaningful information from this complexity. For instance, organisms can selectively respond to certain odorants in odor mixtures (elemental perception). Concurrently, odors can result from the perception of a single percept in multi-component mixtures (e.g., chocolate or coffee aromas, which contain hundreds of odorants [1]) that is different from the quality of each component. When a unique odor is perceived at the expense of the components' odors, the perception is said to be configural. The configural perception is considered to be total when the odors of components cannot be distinguished. It is weak when the odor of the components is perceived together with the specific odor of the configuration [2]. Some mixtures are called “blending mixtures” because they appear to be spontaneously processed, either in a total or in a weak configural way [3]–[7].

Perceptual blending has been especially observed in human adults. In a study in which human subjects were submitted to a typicality rating task, the subjects perceived a binary mixture as smelling like pineapple, while its components carried other odors (strawberry and caramel, respectively [4], [7], [8]). Interestingly, the same binary mixture was also processed configurally in newborn rabbits, which perceived a configural odor on top of the odors of the components in this mixture [5], [9], [10]. This blending property is even more evident in humans with a 6-component mixture, which elicits a typical perception of “Red Cordial” (RC) odor, whereas each single component does not [8]. For adult subjects, in contrast to simpler mixtures (comprising 2 or 3 odorants; [8]), this RC blending mixture seems impenetrable; that is, it is not analyzable in terms of components, even after training with the components at a perceptual or semantic level [7], [8].

Several human studies have underlined the importance of complexity in odor mixture perception [11]–[16], showing that, in mixtures of more than 4 components (4–8 components), the recognition rate of each odorant strongly decreases. Jinks and Laing [11] studied the perception of even more complex mixtures (8–16 components) and noted that subjects who were able to identify 3 very familiar single odorants were unable to identify these familiar odorants once they were contained in a mixture of 16 components. Regarding the number of components, a limit to the elemental perception of odor mixtures by human adults has thus been proposed [13]. This limit does not seem to depend on the subjects' previous experience because it was found even in perfumers or flavorists [14]. These data suggest that a weak configural perception is favored in human adults for mixtures of more than 4 components and that a total configural perception might be the rule for mixtures made of more than 16 components.

The chemical identity of the components is also known to modulate elemental or configural processing of odor mixtures. Thus, several studies reported the impact of key components in the formation of a configural odor, both in human and non-human adult primates [17]–[21]. For instance, hexyl acetate and *trans*-2-hexenal were found to contribute strongly to the formation of an apple odor in a 10-odorant mixture in humans [17]. Similarly, in squirrel monkeys (S*aïmiri sciureus*), the omission of cineole from a learned mixture of 12 odorants led to a decrease in the recognition of the mixture, whereas the omission of another odorant (e.g., linalol or carvone [19]) did not. Large variations in the olfactory perception of given mixtures were also reported as a consequence of modifying the components' proportion in adult rats [2], squirrel monkeys [19] and bees [20], [22], [23]. In human adults, a barely detectable modification of one component's concentration was enough to induce a significant alteration of the pineapple odor quality of a ternary blending mixture [24]. Interestingly, the configural perception of the binary pineapple mixture shifted to elemental perception in newborn rabbits when the proportion of the odorants was also altered [10].

While such factors as complexity, the odorants' chemical identity and the components' proportion have been shown to influence odor mixture perception in various species, no study has dealt with the respective importance of these factors and their generalizability in directly comparing them in two species. This was the objective of the present series of experiments, which were carried out in adult humans and newborn rabbits. Indeed, previous results have shown similarities between these two species in the configural perception of a binary mixture [4]–[10], suggesting a conservation of the processing abilities through evolution. Here, we examined this similarity in the case of more complex mixtures, with the hypothesis that, due to mixture complexity and differences in maturity and between-species sensory capabilities, contrasts might appear. Moreover, a comparison between humans and non-human mammals, even at different ontogenic states, may help improve our general knowledge related to odor processing, and putative conservation and predeterminism of olfactory systems and functions [25], [26].

Here, in these two mammal species, we used the mixture of 6 components (RC) that has been previously shown to elicit the perception of red cordial in human adults [4], [8]. Firstly, the blending property of this mixture was reevaluated in humans with an original approach based on a non-verbal sorting task paired with representations of the results in a perceptual space. This strategy allowed for a straightforward evaluation of the similarity between the mixture and its components. As a control condition, two other mixtures of 6 components were used in order to evaluate their putative configural perception, if one consider the systematic switch from elemental to configural perception suggested for any mixtures with more than 4 components in human adults [11], [13], [14]. Secondly, the contribution of each component and the role of their ratio in the mixture were examined in relation to the global quality of the RC mixture. In rabbit pups, the processing of the RC and the two other 6-component mixtures was evaluated after conditioning to one of their components to assess, for the first time in a young mammal, whether any 6-component mixture is perceived as a configuration. The rabbit pup has the notable advantage of easily learning novel odors with a method of conditioning that has already been validated in the context of perception of binary mixtures [5], [6], [9], [10]. Finally, rabbit pups were conditioned to the RC mixture and tested for their responsiveness to each component to pursue the evaluation of their elemental versus configural perception of the mixture and, in the case of a configural perception, to evaluate whether the perception is completely or weakly configural.

## Materials and Methods

### Ethics statement

To conduct our experiments with human subjects, we followed the Declaration of Helsinki and took into consideration the French laws and regulations applicable at the time the research was conducted (Loi Huriet-Sérusclat, 1988). We did not apply for ethical approval since the experimental design (normal sniffing and sorting of food grade or Pharmacopoeia grade volatile substances at very low concentration on paper strips commonly used in perfumeries) did not fall into the category of the biomedical research, as any design in which “all actions are performed and products used in the usual way, without any additional procedure or unusual diagnostic or monitoring” (Code de la santé publique - lois n°2004-806 du 9 août 2004 et 2006-450 du 18 avril 2006 - Article L1121-1). The participants signed an informed consent form, but the aim of the experiment was not revealed. They were asked to avoid smoking, drinking and eating at least one hour before each session and to avoid using perfume the day of the test. Subjects were paid for their participation (10 €/h). In the animal experiments, we strictly followed the local, institutional and national rules (French Ministries of Research & Technology, and of Agriculture) concerning the care and experimental use of animals. All experiments were conducted in accordance with ethical rules enforced by French law and were approved by the Ethical Committee for Animal Experimentation (Dijon, France) under n° 5305.

### Odor stimuli

The odorants were all purchased from Sigma-Aldrich. The RC mixture was composed of 6 food-grade odorants: vanillin (odorant V; CAS # 8014-42-4), frambinone (F; CAS # 5471-51-2), isoamyl acetate (IA; CAS # 123-92-2), β-ionone (B; CAS # 14901-07-6), ethyl acetate (EA; CAS # 141-78-6), and β-damascenone (D, CAS # 23696-85-7). Stock solutions of all odorants were first prepared at 1% in ethanol (anhydrous, 99.9% Carlo Erba, France). Then, the respective proportion of each stock solution in the mixture was 41.8/41.8/5.0/4.3/4.3/2.8% for V/F/IA/B/EA/D. At this ratio, the RC mixture is perceived by human adults as different from its components, i.e., it smells like red cordial [4], [8].

Four other mixtures were derived from the RC mixture with modification of the components' proportions (the variation did not change the perceived intensity of these mixtures compared to RC; see the section *Preliminary check for iso-intensity* below). Two of these mixtures included relatively weak modifications in the proportion of 2 components: the RC^(V−, IA+)^ mixture contained a proportion of the odorants V/IA that was decreased and increased by 50% (20.9/7.5% of V/IA), and the RC^(B−, EA+)^ mixture contained a proportion of B/EA that was decreased and increased by 50% (2.2/6.3% of B/EA). Two other mixtures presented larger modifications in the components' proportions: the RC^1/6^ mixture included the same proportion of each component (16.7%), and the RC^mod^ mixture included proportions that were entirely redistributed relative to the RC mixture (5.0/4.3/2.8/41.8/4.3/41.8% of V/F/IA/B/EA/D). Six other mixtures were formulated on the same basis as RC but with the deletion of one component, the RC-V, RC-F, RC-IA, RC-B, RC-EA and RC-D mixtures.

Two other mixtures, M^D^ and M^V^, were composed of 6 food-grade components, but all of their odorants except one differed from the RC components. The two mixtures included n-butanol (Nb, CAS # 71-36-3), linalool (L, CAS # 78-70-6), eucalyptol (E, CAS # 470-82-6), α-pinene (P, CAS # 80-56-8), and cis-3-hexen-1-ol (C3H, CAS # 928-96-1). Additionally, the M^D^ mixture included the odorant D (common with RC), while the M^V^ mixture included the odorant V (common with RC). These two mixtures included 16.7% of stock solution of each odorant. In rabbits, stock solutions were prepared at 1% in ethanol for each component. In humans, after checking for iso-intensity (see the section *Preliminary check for iso-intensity* below), the stock solutions were at 30/30/30/10/5/1/1% in ethanol for Nb/P/V/D/E/L/C3H, respectively; they were used both for the constitution of the mixture and the single component stimuli.

In rabbit pups, the mixtures mentioned above were diluted at a final concentration of 10^−5^ g/ml in ultrafiltrated (MilliQ) water (the concentration at which the animals can learn/respond efficiently; see below). Single components were prepared at 1% in ethanol and then diluted at 10^−5^ g/ml in ultrafiltrated water. During behavioral testing, the odorants (alone or mixed) were used at the same concentration as that used during the conditioning. In humans, when the odorants of the RC mixture were used as single stimuli, they were prepared at 10% in ethanol for V, F and EA, and at 1% for B, D and IA, according to the *preliminary check for iso-intensity* between odors (see the corresponding section, below).

### Specific methods for experiments with human adults

#### Participants

Subjects recruited in our laboratory staff (internal panels), including at least 15 judges, participated in a preliminary experiment in order to assess the iso-intensity and pleasantness of the stimuli. Then, three groups of external participants were involved in the main study. They were composed of 73 subjects (G1; 39 women; mean age ± SD: 42.8±15.2 years), 52 subjects (G2; 27 women; 41.7±15.5) and 73 subjects (G3; 34 women; 40.8±13.1) (Table 1). They were considered as “naïve”, that is, they were not exposed to any systematic training in olfaction or sensory analysis. The subjects were recruited with announcements in shops, in newspapers or through a base of volunteers. They were chosen based on a lack of self-reported allergies and problems with their sense of smell. They were asked to avoid smoking, drinking and eating at least one hour before each session and to avoid using perfume the day of the test. Subjects were not aware of either the odors or of the aim of the study.

**Table 1:** Free sorting tasks of human adult participants.

Free sorting task (abbreviation)	Set of odors	Tested hypothesis	Group of subjects
FS1	RC, V, F, B, IA, EA, D	Configural perception (blending effect): *Perception of the RC mixture as compared to its components*	G1 (n = 73)
FS2	Nb, L, E, P, C3H, D, V, M^D^, M^V^	Less configural perception (non-blending effect): *Perception of other 6-components mixtures compared to their components*	G2 (n = 52)
FS3	RC-F, RC-IA, RC-V, RC-EA, RC-B, RC-D, RC	Key components of the blending mixture: *Impact of each component on the perception of the RC mixture*	G2 (n = 52)
FS4	RC^(V−, IA+)^, RC^(B−, EA+)^, RC^1/6^, RC^mod^, RC, RC-V, RC-B	Very specific ratio of components: *Impact of components' proportions on the perception of the RC mixture*	G3 (n = 73)

Set of stimuli, aim and number of subjects involved in each of the 4 free sorting tasks (FS1, FS2, FS3, FS4) (see the Methods section for details on the abbreviations). The same panel of subjects performed FS2 and FS3.

#### Presentation of stimuli

For each mixture or single component, 80 µl of solution were distributed equally on 4 paper strips (20 µl per strip). Then, the strips were let for 2 minutes under an extractor hood to evaporate the ethanol, and then placed into a 60-ml brown glass vial. The different vials were prepared 36 h before testing and kept in the air conditioned room (21°C) where the sensory experiments were carried out. Each set of vials was replaced after testing 10 participants. For a given vial, an interval of at least 1 hour between two successive participants was imposed in order to ensure the gas phase to equilibrate. No difference in perceived intensity was observed between the last and the first participants using the same vials. The vials were coded with a random number.

#### Sensory test procedure

The sensory test consisted of a free sorting task. The task always remained the same, but the odor set to be sorted varied (4 distinct odor sets; Table 1). Subjects were required to smell 7 or 9 stimuli, depending on the odor set. They were told to sort the samples according to their odor similarity. They could make from 1 to 7 or from 1 to 9 groups of stimuli, depending on the odor set (Table 1). They could smell the stimuli as many times as they wanted, but were instructed to wait 20 sec between each stimulus in order to limit adaptation. At the end of the sorting procedure, they were asked to smell the stimuli once again to confirm their sorting before entering their results (groups) in a questionnaire, presented in the FIZZ software (Biosystèmes, Couternon, France). Once their sorting results were recorded, subjects were required to enter a descriptor that best characterized each of the groups that they had constituted, in order to provide a clue to their sorting criterion; they could not, however, further modify their sorting results. To avoid any bias, subjects from group G3, who performed two sorting tasks during the same session (Table 1), were required to record their criterion only after the last task. The order of the two tasks was counterbalanced across subjects.

#### Statistical analysis of sorting data

For each pair of stimuli, the number of subjects that did not gather two stimuli in the same group was considered as a measure of dissimilarity. Thus, symmetric co-occurrence stimulus×stimulus matrices of the total dissimilarities were computed. Dissimilarities were analyzed with Nonmetric Multidimensional Scaling (NMDS; function isoMDS; R software, version 2.10.1, R Foundation for Statistical Computing, Vienna, Austria), a method classically used to analyze free sorting tasks [27]–[29]. This statistical analysis led to the creation of a sensory map, in which the distance between the stimuli represented the perceptual difference as judged by the subjects. The closer two stimuli were on the map, the more often they were grouped together by the subjects and thus were judged to be similar in terms of odor. MDS results depended on the number of selected dimensions kept to map the data. Mapping accuracy was reflected by the stress value of the MDS results, which represents the weighted information loss. A stress value between 10–20% indicates fair, 5–10% indicates good, 2–5% indicates excellent and 0–2% indicates perfect representation of the multidimensional perceptual space; beyond 20%, the representation is considered to be poor [29]. With the use of a bootstrap analysis (5000 draws, R software), a 95% confidence interval relative to the position of each sample in the sensory map was calculated. Mapping was performed using the Matlab® software R2010a (Version 7.10.0.499, The MathWorks, Inc., Natick, MA).

#### Preliminary check for iso-intensity between odor stimuli

To evaluate whether human subjects perceived differences in intensity between the stimuli used in each sorting task, iso-intensity was checked for each odor set (Table 1) with the help of internal panels (15–23 subjects). These panelists were required to evaluate each stimulus intensity on a 10-cm linear scale without graduations (end point anchors: low intensity vs. high intensity). The distance in cm from the origin of the scale and the mark provided by the subject was considered as reflecting the intensity and was thus used as the variable [7], [17]. Means were further calculated across subjects. Stimuli were presented in a counterbalanced order across subjects.

Regarding stimuli included in the FS1 odor set (RC mixture and its components; see Table 1), a pairwise comparison of means (paired t-test) revealed no difference in intensity (1.82>t[14]>0.06; 0.09<p<0.96), with the exception of EA, which was perceived as less intense than the other odorants, except for F (3.38>t[14]>2.47; 0.004<p<0.019). Concerning the stimuli from the second odor set (FS2), subjects evaluated the components of the M^V^ and M^D^ mixtures as generally iso-intense (P, L, E, C3H, Nb, D); however, odorant D was perceived as less intense than M^D^ (t[14] = 2.91, p = 0.01), and odorant V was perceived as less intense than all other stimuli (5.24>t[14]>2.25, 0.0001<p<0.04). Regarding the FS3 odor set (RC-X and RC mixtures; Table 1), all stimuli were considered to be iso-intense (1.84>t[14]>0.02, 0.09<p<0.98). Finally, the RC, RC-V and RC^mod^ stimuli from the FS4 odor set were rated as iso-intense; however, RC^(B−, EA+)^ was judged as slightly more intense than RC^1/6^, RC^mod^ and RC-V, and RC^1/6^ appeared to be less intense than RC (4.21>t[22]>2.36, 0.0003<p<0.03). Nevertheless, none of these slight differences in intensity accounted for the sorting results. Indeed, for more than 69% of the subjects, the freely chosen sorting criteria were related to odor quality, while less than 27% were related to intensity and less than 0.1% were related to pleasantness.

### Specific methods for experiments on rabbit pups

#### Animals and housing conditions

Male and female New Zealand rabbits *Oryctolagus cuniculus* (Charles River strain, L'Arbresle, France) were bred in the Centre de Zootechnie (UB, Dijon) as previously described [5], [6], [9], [10]. A nest-box was appended on the outside of the pregnant females' cages 2 days before delivery [day of delivery was postnatal day (PND) 0]. After parturition, females had daily access to their nest between 11:30–11:45 a.m. This procedure allowed for females to follow the brief (3–4 min) daily nursing of the species [30], and for experimenters to assess that pups sucked efficiently. A total of 382 pups born from 99 females were used in the study.

#### Odor conditioning and behavioral assay

The procedures were run as described in detail by Coureaud and colleagues [5], [9], [10], [31] and Sinding and colleagues [6]. The Mammary Pheromone (MP; 2-methylbut-2-enal, CAS 6038-09-1) has the property to induce the learning of single odorants or of mixtures of odorants through associative conditioning (when used at 10^−5^ g/ml). On PND 1, the pups were conditioned for 5 min with the use of a cotton pad scented with the MP+odorant or MP+mixture solutions. At the end of the conditioning, the pups were individually marked with scentless ink and returned to their nest. Only 4 or 5 pups from the same litter contributed to a given group (the remaining pups in the litter were entered into another group). All of the conditioning sessions were performed 1 h before the daily nursing episode (at 10:30 a.m.) to equalize the pups' motivational state and limit the impact of satiation on their responsiveness [32].

On PND 2, 24 h after the conditioning, the pups were tested in an oral activation test. The assay consisted of the presentation of the stimulus in front of the nares of each pup with a 0.5-cm glass rod for 10 sec. A test was positive when the stimulus elicited typical orocephalic searching movements, usually followed by grasping movements (oral seizing of the rod). Non-responding pups displayed sniffing but no oral seizing.

Each pup participated in only one experiment but was successively tested with 2–4 stimuli, depending on the experiment (no more than 4 to avoid fatigue or habituation; the order of stimuli presentation was systematically counterbalanced across pups; interval between two stimulations: 120 s). Each experimental group was drawn from 4 to 7 litters in order to minimize litter effects [9], [10].

#### Statistical analyses

The pups that did not respond to any of the tested stimuli were not considered to be conditioned, and they therefore were not included in the analysis (n = 42, equally distributed across litters). The proportions of the responding pups were compared using Pearson's χ^2^ test when the groups were independent (i.e., different pups tested for their responsiveness to the same stimuli) or the Cochran's Q test when the groups were dependent (i.e., same pups tested to different odorants). When the Cochran's Q test was significant (multiple comparisons), the proportions of responding pups were compared 2×2 by the McNemar χ^2^ test. The Pearson and McNemar χ^2^ tests were used with Yate's correction when necessary. Degrees of freedom are indicated when >1. Comparisons between data were considered to be significant when the two-tailed test yielded a p<0.05.

## Results

### Experiments with human adults

#### Perception of the RC mixture compared to its components

**Figure 1:**
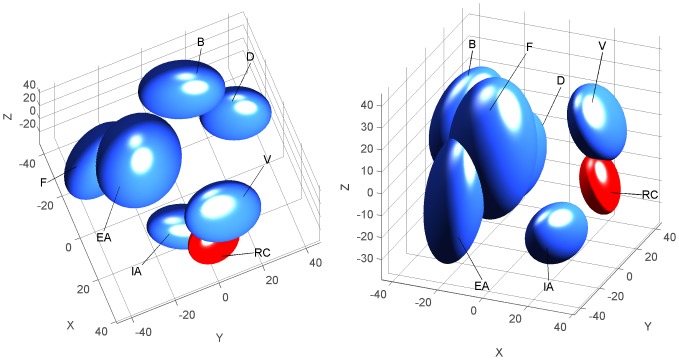
Human perception of the RC mixture. 3D representation (2 different views) of the sensory map obtained from the free sorting task FS1 based on the sorting of the RC mixture and of its components (EA, D, V, IA, B, F) by human adults. The map was drawn using a non-metric MDS analysis. Ellipsoids represent 95% confidence intervals and were calculated using a bootstrap algorithm.

To compare the perceived odor quality of the 6-component RC mixture with the odor quality of its individual components, subjects were asked to sort both the mixture and the components (FS1; Table 1). The results were reported on a sensory map, presented in Figure 1. With 3 dimensions (3D), the stress value of the representation was 2.99% (0.002% with 4D). In the 3D sensory map, 95% confidence intervals on the position of each stimulus are represented by ellipsoids (Figure 1). Non-overlapping confidence intervals evidenced a good separation between RC and 4 of its components (F, EA, B, D). The 4D representation confirmed that confidence intervals around RC did not overlap with any of its components; RC was particularly well separated from component V. In contrast, confidence intervals overlapped between odorants F and EA, indicating that these 2 odorants were perceived to be relatively similar. The small size of the RC ellipsoid compared to those of its components underlined a good agreement between subjects concerning their relative perception of RC compared to its components.

Thus, the representation in a perceptual space of the subjects' perception illustrated a clear difference between the odor of the RC mixture and the odors of its components. This result confirmed the emergence of a configural odor in RC for human adults.

#### Perception of the other 6-component mixtures compared to their components

**Figure 2:**
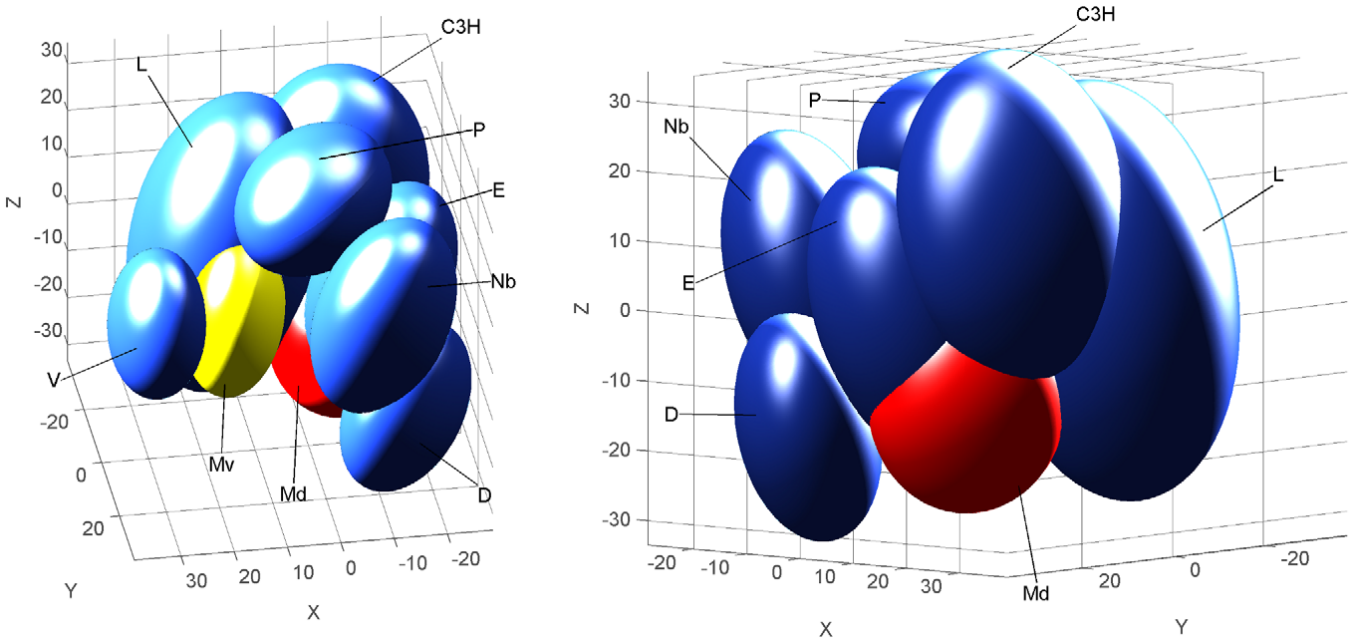
Human perception of the M^V^ and M^D^ mixtures. 3D representations (2 different views) of the sensory map obtained from the free sorting task FS2 based on the sorting of two 6-component mixtures (M^V^ and M^D^) and of their components (L, E, Nb, C3H, P, V and D) by human adults. The map was drawn using a non-metric MDS analysis. Ellipsoids represent 95% confidence intervals and were calculated using a bootstrap algorithm.

To assess whether any mixture of 6 components carried an odor quality that was distinct from the quality of its components, other subjects were engaged in a sorting task that included two other mixtures (M^V^ and M^D^) and their odorants (FS2; Table 1). The stress value associated with the 3D representation was 7.7%, which is “acceptable” (see the Methods section; 2.6% with the 4D representation). Contrary to the sensory map obtained with the RC mixture and its components (Figure 1), the results of FS2 produced very aggregated and overlapping confidence interval ellipsoids for almost all samples (Figure 2); the M^D^ and M^V^ mixtures were closely surrounded by all their components. Moreover, in 4D and 3D representations, the confidence intervals of M^D^ overlapped with those of odorants E, C3H and L, and those of M^V^ overlapped with L; the confidence intervals of odorants L and C3H were also overlapping.

Thus, any mixture of 6 components compared to its components did not lead to a sensory map comparable to RC (Figure 1). Here, the M^D^ and M^V^ mixtures appeared to be categorized with some of their odorants, highlighting a perceptual similarity between the mixture and the components.

#### Impact of each component on the perception of the RC mixture

**Figure 3:**
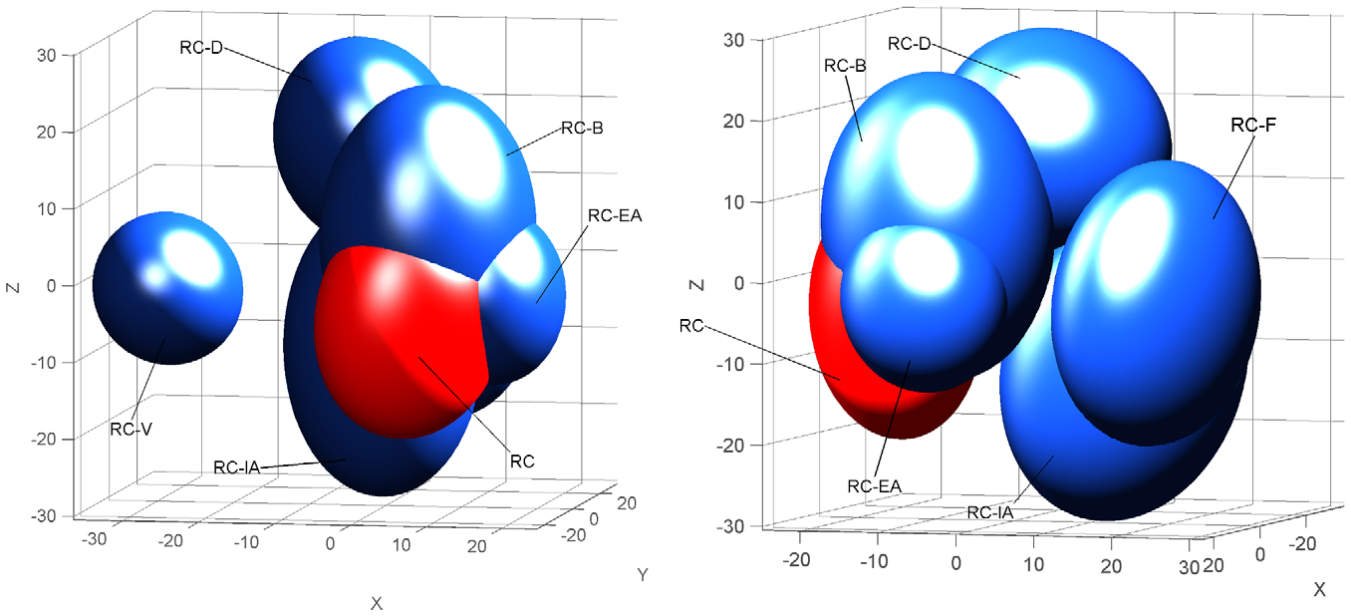
Human perception of the RC vs. RC-X mixtures. 3D representation (2 different views) of the sensory map obtained from the free sorting task FS3 based on the sorting, by human adults, of the RC mixture and 6 RC-X mixtures (formulated as the RC mixture but deleted of one component). The map was drawn using a non-metric MDS analysis. Ellipsoids represent 95% confidence intervals and were calculated using a bootstrap algorithm.

To evaluate the relative impact of each odorant on the perception of the RC mixture's configuration, subjects were required to sort seven stimuli constituted by the 6-component RC mixture and six mixtures of 5 components identical to RC, from which one component was omitted (RC-X) (FS3; Table 1). The stress value associated with the 3D representation of the sorting results was extremely low (0.007%) and was therefore considered to be “good.” The distribution of the confidence interval ellipsoids revealed that RC overlapped with RC-EA and RC-B (Figure 3). On the other hand, RC-V was well separated from all other mixtures and was the most distant mixture from RC. Thus, the RC mixture did not appear to be equidistant from all of the mixtures from which one odorant was omitted. RC was thus likely perceived as less different from RC-EA and RC-B than from RC-V and RC-F.

#### Impact of components' proportion on the perception of the RC mixture

**Figure 4:**
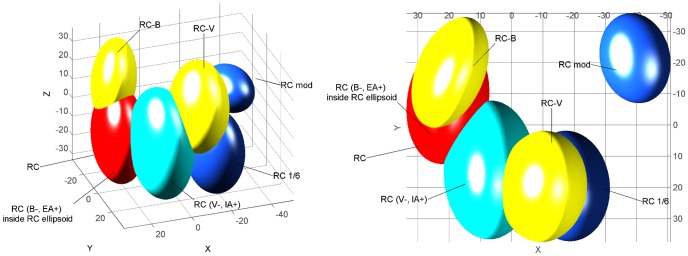
Human perception of RC mixtures varying in component's proportions. 3D representations (2 different views) of the sensory map obtained from the free sorting task FS4 based on the sorting, by human adults of the RC mixture and several mixtures formulated as RC but with varying proportions of two components (RC^(B−, EA+)^ and RC^(V−, IA+)^ (light blue), equalized proportions of odorants at 16.7% (RC^1/6^, dark blue), redistributed components proportion (RC^mod^; dark blue), omission of odorant V or B (RC-V and RC-B, yellow). The map was drawn using a non-metric MDS analysis. Ellipsoids represent 95% confidence intervals and were calculated using a bootstrap algorithm.

To test whether specific proportions of components are required to promote the perception of a configuration in the RC mixture, subjects were required to sort 7 stimuli consisting of the following: the RC mixture; 4 variants in which the proportions of only two [RC^(B−, EA+)^ and RC^(V−, IA+)^] or all of the components (RC^1/6^ and RC^mod^) were modified; and 2 mixtures, identical to RC, but in which one component was omitted (RC-V and RC-B) (FS4; Table 1). The last two stimuli allowed for the comparison of the respective impacts of proportion modification and of component omission. The stress value associated with the 3D representation of the sensory map drawn from FS4 results was only 0.009% (Figure 4). Evaluating the confidence intervals, the RC^(B−, EA+)^ mixture completely overlapped with RC (i.e., it is masked by RC on Figure 4) and partially with RC^(V−, IA+)^. On the contrary, RC^1/6^ and RC^mod^ mixtures were well separated from RC. RC was more distant from RC^mod^ than from RC^1/6^, and RC^mod^ was well separated from all other mixtures. Regarding the relative impact of the component's proportion versus the component's omission, RC^mod^ was more distant from RC than RC-V, the confidence interval of RC^1/6^ overlapped with RC-V, and RC-V was farther from RC than RC-B, while RC-B overlapped with RC.

These results underlined that the proportion of the odorants influenced the mode of perception of the RC mixture, and it especially impacted the emergence of an odor quality that was specific to the mixture (i.e. the configuration). The more the components varied in proportion, the more the quality of the mixture was modified. Moreover, the ratio of odorants seemed to have a greater impact on the RC perception than the omission of one component.

### Experiments with rabbit pups

#### Perception of the RC mixture after learning of one of its components

**Figure 5:**
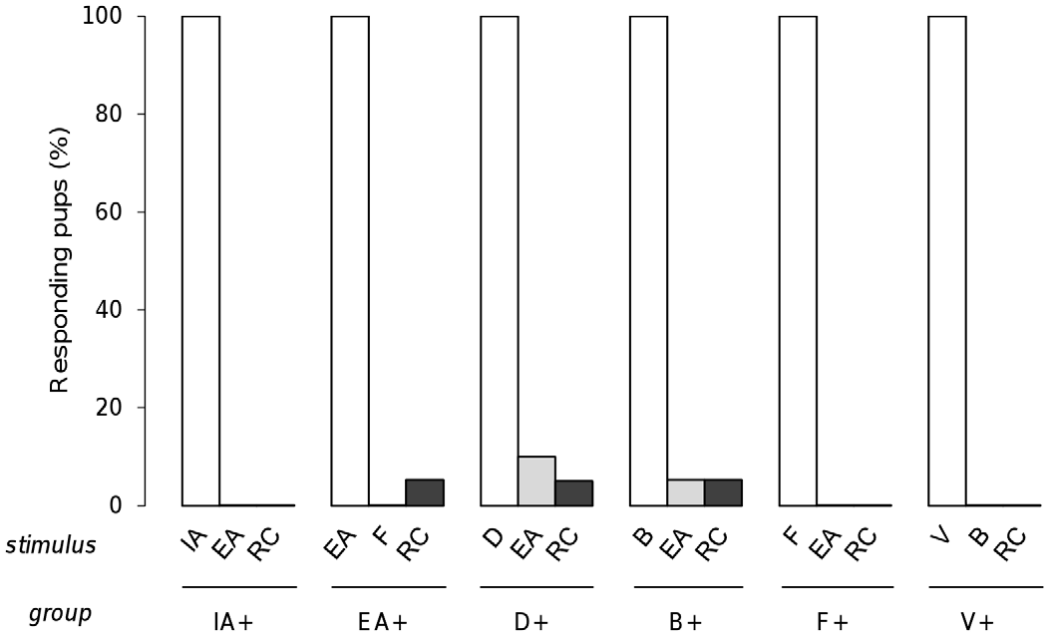
Rabbit perception of the RC mixture. Proportions of 2 day-old rabbit pups responding in an oral activation test, after conditioning to one odorant of the RC mixture (IA+, EA+, D+, B+, F+ or V+), to the odorant they were conditioned to (white bars), to another unfamiliar component from RC (grey bars) and to the RC mixture (black bars) (n = 16, 19, 20, 19, 13 and 16; respectively in IA+, EA+, D+, B+, F+ and V+).

To assess whether the RC mixture was processed by rabbit pups in a configural way, 6 independent groups of neonates were tested for their responsiveness to RC after conditioning to only one component, i.e. after conditioning to odorant IA (16 pups from 5 litters), EA (19 pups, 5 litters), D (20 pups, 7 litters), B (19 pups, 5 litters), F (13 pups, 4 litters) or V (14 pups, 5 litters). During the assay, the pups were also tested with the conditioned odorant and with one non-conditioned odorant that was chosen among the other RC components. On one hand, the results revealed that all pups from all the groups (100%) responded to the component to which they were conditioned and not, or very infrequently, to the non-conditioned odorant (<11%; conditioned vs. non-conditioned odorant: χ^2^>11.7, p<0.001; non-conditioned odorants between them: χ^2^<0.38, p>0.53 in all comparisons). On the other hand, the pups' responsiveness to RC was always null or negligible, regardless of the group (<5.3%; χ^2^<0.03, p>0.85 for between groups comparisons), and then was highly different compared to the responsiveness to the conditioned odorant (χ^2^>11.7, p<0.001 in the different comparisons) (Figure 5).

Thus, after learning a single, initially neutral component, the pups responded to this odorant, but they did not generalize the learned information to the RC mixture.

#### Perception of the other 6-component mixtures

**Figure 6:**
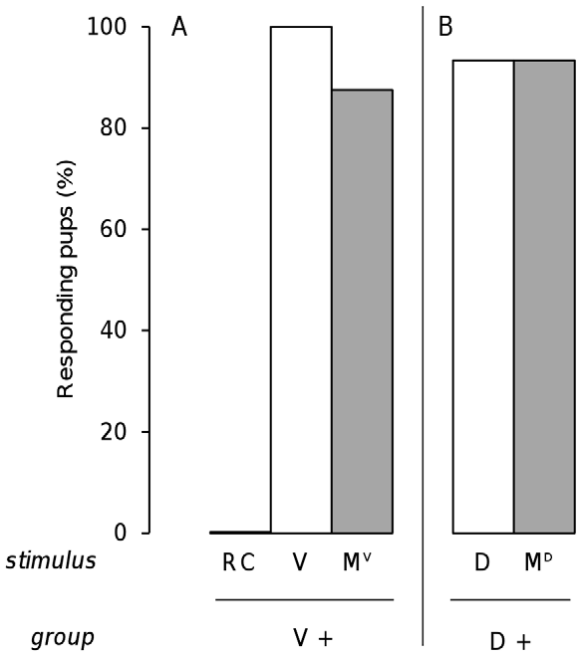
Rabbit perception of the RC vs. M^V^ and M^D^ mixtures. Proportions of 2 day-old rabbit pups responding in an oral activation test A) to the RC mixture, the component V of this mixture (white bar) or to another mixture of 6 components including V (M^V^; grey bar), after conditioning to the odorant V (group V+; n = 16); or B) to the component D of the RC mixture (white bar) or to another mixture of 6 components including D (M^D^; grey bar), after conditioning to the odorant D (group D+; n = 15).

The absence of pup response to the RC mixture after learning one of its components could be due to their perception of an RC configuration, i.e., of a novel odor that emerged specifically from the mixture formed by these components. However, it could also result from the configural processing of any mixture that becomes too complex (as in humans with mixtures containing more than 3 components, [11]–[13]). To address these hypotheses, rabbit neonates were tested for their responsiveness to the M^V^ and M^D^ 6-component mixtures, which should not blend, after conditioning to one of their components. Thus, a group of pups was conditioned to odorant V and tested to odorant V and to mixtures M^V^ and RC (16 pups, from 4 litters). The day after conditioning, all pups responded to V, 87.5% responded to M^V^ and no pups responded to RC (M^V^ vs. V: χ^2^ = 0.5, p = 0.48; RC vs M^V^ or V: χ^2^>12.07, p<0.001). Another group was conditioned to odorant D and tested to odorant D and to the M^D^ mixture (15 pups, 4 litters). After conditioning to D, the pups strongly and indifferently responded to D and to M^D^ (respectively 93.3 and 87.5%; χ^2^ = 0.5, p = 0.48). The respective responsiveness to M^V^ and M^D^ of the pups conditioned to V or D was similar (χ^2^ = 0.21, p = 0.64) (Figure 6).

Thus, the lack of response to the mixture after learning one of its components was only observed for RC. Conversely, in the same learning situation, pups responded to the other mixtures of 6 components.

#### Impact of each component on the perception of the RC mixture

**Figure 7:**
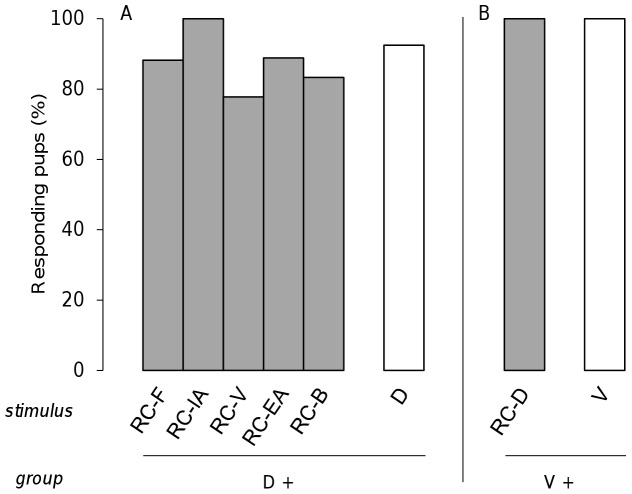
Rabbit perception of the RC vs. RC-X mixtures. Proportions of 2 day-old rabbit pups responding in an oral activation test A) to the RC mixture deleted of one component (RC-X where X represents the removed component; grey bars) and to the component D from the RC mixture (white bar) after conditioning to the component D (results were pooled from 3 groups of pups; n = 53); or B) to the RC mixture deleted of the component D, and to the component V from the RC mixture (white bar) after conditioning to the odorant V (one group; n = 20).

To evaluate whether each component of the RC mixture contributed equally to the neonatal perception of the RC configuration, the pups were conditioned to one component of RC before being tested to different RC mixtures in which another component was deleted. Three groups of pups were thus conditioned to odorant D and tested either to RC-F, RC-IA and D (17 pups, 5 litters), to RC-V, RC-EA and D (18 pups, 5 litters), or to RC-B and D (18 pups, 5 litters). During the test, most of the pups responded to D (92.5%). In addition, their responsiveness was high and similar for all of the RC-X mixtures (range: 77.8–88.9%; Q<3.0, ddl = 2, p>0.22 and χ^2^<1.81, p>0.18 for all dependent and independent comparisons) (Figure 7). Moreover, to determine whether these results appeared for any conditioned odorant, another group of pups was conditioned to odorant V and tested to RC-D and to V (20 pups, 5 litters). They all responded (100%) both to V and to RC-D (Figure 7).

In sum, the deletion of one component in the RC mixture was followed by a strong responsiveness of the pups to the RC-X mixtures, regardless of the omitted component.

#### Impact of components' proportion on the perception of the RC mixture

**Figure 8:**
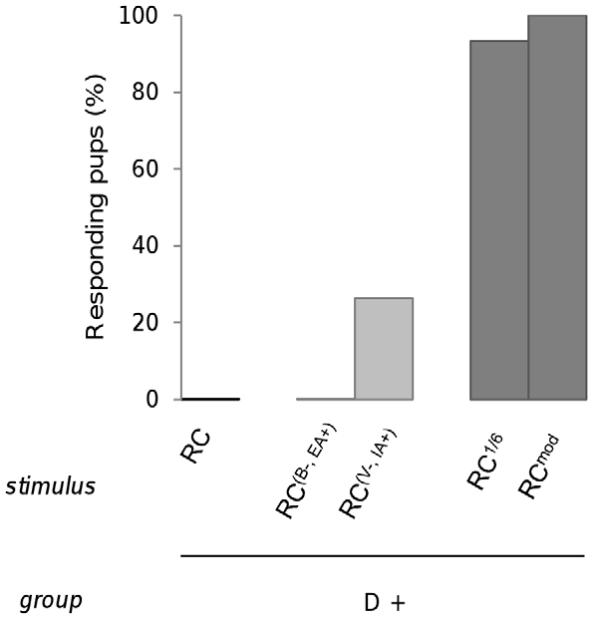
Rabbit perception of RC mixtures varying in component's proportions. Proportions of 2 day-old rabbit pups responding in an oral activation test, after conditioning to the odorant D of the RC mixture, to the RC mixture and to four mixtures derived from the initial composition of RC in terms of proportions of components: the RC^(B−, EA+)^, RC^(V, IA+)^, RC^1/6^ and RC^mod^ mixtures (results were pooled from 3 groups; n = 47).

To determine whether the non-response to RC after learning one component depends on its composition in terms of odorant proportion, 3 groups of pups were conditioned to odorant D and then respectively tested to RC and RC^(B−, EA+)^ (13 pups, from 4 litters), to RC and RC^(V−, IA+)^ (19 pups, 6 litters) or to RC^mod^ and RC^1/6^ (15 pups, 4 litters). During the assay, no pups responded to RC (as in our first rabbit experiment, see above). They also did not respond to RC^(B−, EA+)^. However, 26.3% of them responded to RC^(V−, IA+)^, a proportion that tended to be higher compared to that observed for RC (χ^2^ = 3.2, p = 0.07). Moreover, their responsiveness was strong to both RC^1/6^ (93.33%) and to RC^mod^ (100%) and was clearly different from that observed for RC^(B−, EA+)^ and for RC (χ^2^>20.6, p<0.001) (Figure 8).

Thus, modifying the proportions of certain components within the RC mixture improved pup responsiveness to the mixture: more radical changes resulted in higher improvement.

#### Neonatal ability to learn and to elementally process the RC mixture

**Figure 9:**
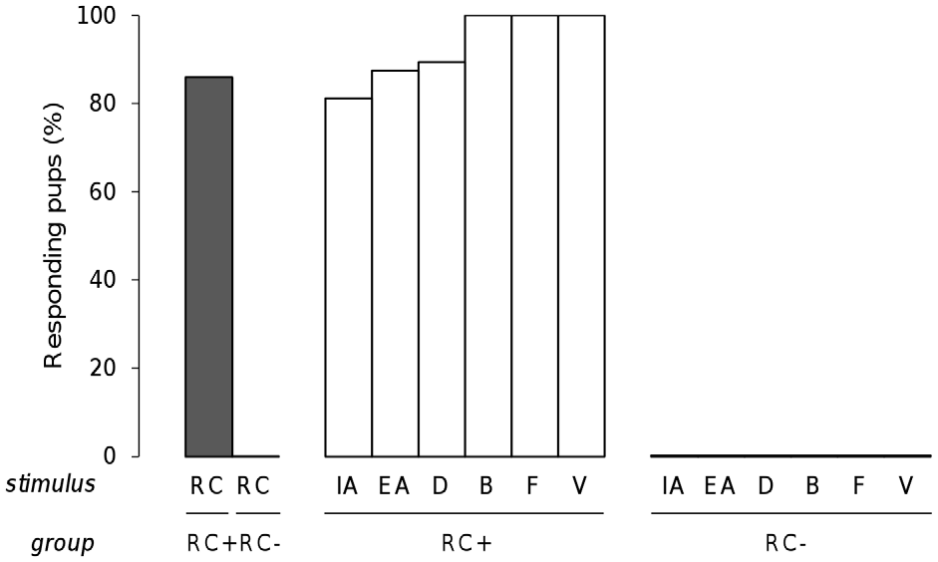
Elemental perception of the RC mixture by newborn rabbits. Proportions of 2 day-old rabbit pups responding in an oral activation test to the RC mixture (black bar) and to its components (white bars) after 1 conditioning to RC (RC+) or in absence of conditioning to RC (RC−) (n = 54 and 25, respectively).

The above experiments indicate that, after being conditioned to one component of a mixture, newborn rabbits process the 6-component RC mixture configurally. But are they completely unable to perceive one or several elements in the RC mixture? To address this question, other pups were conditioned to RC and tested later for their responsiveness to RC and odorants IA and EA (16 pups, from 5 litters), RC and odorants B and D (19 pups, 5 litters), or RC and odorants F and V (19 pups, 5 litters). In addition, 25 control pups (3 litters) were tested for their responsiveness to RC and to either IA and EA (n = 9), to B and D (n = 8) or to F and V (n = 8), without any previous conditioning to the mixture. While none of the pups responded to RC in the absence of conditioning, 85.2% responded to it after conditioning (results pooled from the three conditioned groups; χ^2^ = 47.54, p<0.001). Interestingly, after conditioning to the RC mixture, a high and similar proportion of pups also responded to its components when presented separately (range: 81.5–100%; χ^2^<1.87, p>0.17 in all the comparisons). The pups then responded strongly both to RC and to its components (χ^2^<3.2, p>0.05) (Figure 9).

Thus, rabbit pups were able to rapidly learn the complex mixture of 6 odorants and to later respond to not only the mixture but also to its elements.

## Discussion

The present experiments aimed to study the perception of a complex mixture, the red cordial (RC) mixture, which induces perceptual blending in humans. The perception of this RC mixture, studied here both in human adults and newborn rabbits, presents several striking similarities between the species in our conditions. Rabbit pups, like human adults, perceived the odor of the mixture to be distinct from the odor of its components. However, in both species, this configural perception was not observed for any mixture of 6 odorants. Moreover, the omission of any one component from RC disrupted the configural processing in the rabbits. In humans, the impact of such omission appeared to be more subtle, as the removal of certain components altered the configural perception more than the removal of others. Furthermore, the proportion of odorants in the mixture strongly determined the way in which the mixture was perceived in both species. The configural perception specific to the blending effect was immune to a certain level of proportion variation; however, after large modifications, the mixture was no longer perceived as a configural odor but as some of its components. In spite of these inter-species similarities, newborn rabbits could separate the RC mixture into its elements after learning the mixture. Rabbit pups might then express higher abilities for elemental perception than human adults. Indeed, a limit in elemental processing of odor mixtures has been pointed around 4 components in humans [11], [13]. Such a limit remains to be tested for the RC mixture that we presently used.

### 

#### Perceptual blending effect in the RC mixture

In humans, the blending property of the RC mixture was confirmed using a new method based on a free sorting task paired with 3D representation of the perceptual space corresponding to the odor set. A comparison of the 3D odor space representation obtained for RC relative to those obtained for 2 other mixtures (M^V^ and M^D^) revealed clear differences. Indeed, RC was separated from all its components, whereas M^V^ and M^D^ were not significantly separated from some of their odorants. As we found that the sorting mainly relied on odor quality categorization, it can be concluded that the RC odor was perceived to be different from the odors of all the RC components, confirming configural perception. These results even suggest the “complete” (also named “robust”, see [2]) configural perception of RC. On the contrary, the M^V^ and M^D^ mixtures appeared to be perceived elementally, at least in part, because the odors of some of the components were still perceived within the mixture. With the present method, the perception of a configural odor in these mixtures, in addition to the perception of certain elements, cannot be excluded. Concerning the M^V^ and M^D^ mixtures, one may note that their complexity (6 odorants) could induce a loss of perception of some, but not all, of the components. This is in agreement with the suggestion that in human adults, some mixtures that include more than 4 components would retain certain specific qualities of the odorants on top of the putative emergence of a novel odor [12].

In terms of method, the use of a free sorting task was particularly relevant in order to reveal the configural perception of the RC mixture, as it reflected the actual perceptual similarity (or dissimilarity) between the mixture's odor (configuration) and the odors of its components. It has been shown that human categorization of odors is based on perceptual similarity and is in part independent of linguistic skills [33]. In previous studies [4], [8], the configural perception of RC was revealed by a typicality test that aimed to evaluate the matching between a concept, “red cordial” in the case of the RC mixture, and the mixture's (or single components') actual odor. This test repeatedly ended in a higher RC typicality for the mixture compared to its components. However, it did not offer any information on the possible perception of elements in RC in addition to the configuration, that is, of the weak versus complete configural perception of the mixture. Taken together, the results of both methods (sorting and typicality) clearly evidenced that the RC mixture i) carries an odor quality that is significantly different from the odors of its components and ii) evokes a red cordial odor, which was not carried by its constituting odorants. These results demonstrate that human adults perceive the RC mixture as a configuration, validating the blending property of this mixture for adults of our species (at least in cultures in which it has been tested: here, France, and Australia in [4]).

Interestingly, newborn rabbits also seemed to perceive a configuration in RC, as they did not respond to the mixture after learning of one of its odorants. This lack of response was not simply due to the presence of unfamiliar odorants in the mixture in addition to the learned component because rabbit pups strongly responded to other mixtures of 6 components (M^V^, M^D^) after learning the same odorant. Moreover, the absence of response to RC was not a consequence of any masking of the learned odorant by one or several of the other odorants because this lack of response was observed for each odorant learned by the pups. Thus, for rabbit pups, the RC mixture presents a specific perceptual property that leads to the weak perceptual fusion of the components' odors and to the final perception of a new odor when these components are combined in the mixture. The specific configural RC odor quality would then prevent the pups' response to the learned odorant when encountered in the mixture. Such a configural effect had already been highlighted in rabbit pups with a less complex binary mixture that smelled like pineapple to humans [5], [6], [9], [10].

#### Contribution of each odorant to the configuration in the RC mixture

To address that point both in humans and rabbits, we assessed the perception of the RC mixture after removal of one of its components. In humans, the perception of the resulting 5-component mixtures differed from the 6-component RC mixture, according to the nature of the omitted component. Indeed, when vanillin (V) or frambinone (F) was removed from RC, the global odor of the mixture was more affected than after omission of ethyl acetate (EA) or β-ionone (B), suggesting a lower contribution of these last 2 odorants to the configuration. Compared to frambinone, vanillin seemed to be more involved in the configural perception of RC because the RC-V mixture was evaluated as more dissimilar from RC but also from all other mixtures in the perceptual space. This is also supported by the result showing that vanillin's odor was perceptually close to the odor of the RC mixture.

In rabbit neonates, the removal of one odorant from RC systematically led to high responsiveness to the 5-component mixture after conditioning to one of its components. Such responsiveness was not displayed for RC itself. This difference did not result from a general switch from elemental to configural perception when the number of odorants in mixture increases from 5 to 6 because rabbit pups perceive other mixtures of 6 odorants (M^V^ and M^D^) in an elemental way. Again, it seems that this difference results from the blending property of RC, that is, from the emergence of a configural odor due to the mix of these 6 particular odorants. Moreover, the pups' responsiveness to the RC-X mixtures was always high and similar, regardless of the odorant deleted. Thus, the various components of RC apparently contribute equivalently to the perception of the RC configuration by newborn rabbits. These results therefore suggest that all 6 odorants are strictly necessary for the RC configuration to be perceived. Nevertheless, the nature of the paradigm used to test the newborn rabbits' responsiveness and the nature of their response (on/off response) may be insufficient to discriminate between subtle variations in the perception of the odorants, as observed in our study with human adults but also in other studies with insects. In honeybees (*Apis mellifera ligustica*), following an appetitive conditioning to 2 floral mixtures of 6 components, certain individuals, named “selective learners” (in opposition to “non-selective learners” and “non-learners”), did not respond uniformly to all the components, but more selectively to 3 of them, which were defined as key-odorants [18]. These differences among bees might result from genetic determinism or from previous experience with the 3 odorants in the environment. Another study in bees showed that they refer to a sub-mixture of key-odorants in order to discriminate between 3 floral mixtures of 14 odorants, one specific to each floral mixture; these key-odorants are the most concentrated in the mixture [20].

#### Impact of odorants' proportion on the configural perception of the RC mixture

In humans, modifying the proportion of 2 odorants in the RC mixture [RC^(B−, EA+)^, RC^(V−, IA+)^] was not clearly followed by a distinct perception of these mixtures relative to RC. The RC^(B−, EA+)^ mixture was so similar to the original RC mixture that the RC ellipsoid included the RC^(B−, EA+)^ ellipsoid, whereas the RC^(V−, IA+)^ ellipsoid only partly overlapped with RC ellipsoid (Figure 3), likely because of the key-odorant status of vanillin (V), as previously suggested. In contrast, the modification of all components' proportions (RC^1/6^ and RC^mod^) prevented the perception of the configural odor. Therefore, the combination of specific odorants was not sufficient to induce the configural processing of the RC mixture; this processing also depended on the relative proportions of these components. In that regard, the present results correlate with the results obtained with a binary and ternary blending mixture in human adults, indicating that a variation in the concentration of one component can induce a significant modification of the specific odor quality of the mixture [4].

After conditioning to one component of the RC mixture, the rabbit pups did not respond to the RC^(B−, EA+)^ mixture. This suggests a configural perception of both mixtures and a perceptual similarity between these mixtures, as suggested in human adults (RC and RC^(B−, EA+)^ completely overlap on the map resulting from the human sorting task). Conversely, in rabbits as in humans, the response to the RC^(V−, IA+)^ mixture tended to be somewhat different from the response to the original RC mixture, a result that could support a stronger impact of V and IA in the configuration compared to B and EA (but see the above discussion of omission). After a strong modification (RC^mod^) or equalization (RC^1/6^) of the proportions of all components, most of newborn rabbits responded to the modified mixtures, which demonstrated their elemental perception of these 6-component mixtures and their ability to respond to the learned elements that they contained. A previous study showed similar results after modification of the ratio of components in a binary blending mixture [10]. In sum, for rabbit neonates as well as for human adults, the configural perception of RC strongly depends on the components' proportions as well as their chemical identity.

#### Elemental abilities of newborn rabbits

In the present study, after a single and short conditioning to the RC mixture, the rabbit pups responded strongly to each single component, without differences between components. Thus, the pups were able to detect all of the elements of the mixture during the conditioning, and they were also able to memorize and retain them during at least 24 hours. This suggests that the RC mixture may be processed in a weak but not a completely configural way, meaning that pups would perceive a configural odor in the mixture in addition to perceiving the odors of the components. From this perspective, these results correlate with those observed in rabbit neonates concerning their weak configural processing of a binary mixture, which also induces perceptual blending in human adults (mixture smelling like pineapple, [5], [6], [9], [10]).

These results show that newborn rabbits present some ability to detect the elements (odorants) contained in the RC mixture. Regarding human adults, the RC mixture was sorted as clearly distinct from its components, indicating a poor similarity between the mixture and its elements and suggesting that human adults could not perceive the components within the complex odor mixture, as discussed by other authors [11]–[13]. To our knowledge, the high analytic capabilities displayed here by rabbit pups have not often been reported in the literature. Only one study demonstrated that after training to a 6-odorant mixture, honeybees recognize and respond to each component [18]. We suggest that the differences observed between the elemental abilities of rabbit neonates and human adults result from differences in the experimental paradigms: MP-induced conditioning could confer a strong biological value to all associated stimuli (mixture or odorants) because of the high biological value of the MP. More generally, the reinforcing effect of the MP could favor the simultaneous memorization of several odor signals and their long-term retention. In contrast, in the human protocol, subjects were not engaged in an associative conditioning procedure; thus, they were not subject to the deep modification of odor percept representation that is enforced by such procedures [34]–[36]. For example, odorants similar in perceived quality could be discriminated in a motivated discrimination task [34]. Similarly, human subjects acquire the ability to discriminate between enantiomers when one is reinforced by aversive conditioning (electric shock [37]). Therefore, one may suggest that the processing of the RC mixture could become elemental if human subjects are submitted to experimentally controlled conditioning and also, more generally, that odor mixture processing is modulated by motivational or attentional effects. This remains to be demonstrated with dedicated experiments.

Apart from these experimental considerations, certain biological factors may also explain the strong elemental abilities of newborn rabbits. First, these differences could be related to the reduction in the number of genes dedicated to olfaction in primates (396 in humans) compared to other mammals (e.g., 552 in rabbits, 1259 in rats; see the Sevens Database [38] and [39]). However, it has been shown that despite such genetic differences humans are “good smellers” [40]. For instance, humans performed as well as squirrel monkeys and as well or slightly better than dogs and rats when tested for thresholds to the odors of a series of straight-chain aldehydes [41]. Nevertheless, to date, no study has evaluated the abilities of organisms to analyze elements in mixtures according to the olfactory receptor repertoire. Secondly, elemental abilities may depend on working memory [42]. Odor mixture processing is a dynamic process, implying the temporal coding of odorants [42], [43]. In humans, it has been estimated that the working memory involved in odor identification operates in approximately 700 ms [44]. Therefore, the slow processing of working memory could prevent the recognition of each odorant within the mixture and may thus account for the limited capacity of humans to elementally process complex odor mixtures [42]. Dedicated experiments are required to confirm this hypothesis. Finally, elemental abilities of rabbit pups may result from their developmental stages and ecological constraints. Indeed, they are devoid of functional vision and audition and are fully dependent on olfaction to survive (e.g. [45]–[47]). Their olfactory brain may thus be especially competent in processing odorants in mixtures. More generally, in very young organisms, the urgent need to acquire knowledge about the novel, initially relatively limited environment may result in high elemental abilities that allow to process, detect, learn and respond to a wealth of information from the surroundings; thus, immediate adaptation to the environment could be optimized, as anticipation of its inevitable changes.
